# The quantitative detection method employing a combination of high-affinity antibodies targeting PCT^☆^

**DOI:** 10.1016/j.bbrep.2025.102091

**Published:** 2025-06-30

**Authors:** Xiaoxia Cheng, Lichen Zha, Jiao Yang, Yinyin Qin, Ruhong Yan, Yuzhu Ma, Changsong Zhang, Hongran Fu

**Affiliations:** a Department of Clinical Laboratory, Suzhou Hospital, Affiliated Hospital of Medical School, Nanjing University, Suzhou, Jiangsu, China; b Suzhou Research Center of Medical School, Suzhou Hospital, Affiliated Hospital of Medical School, Nanjing University, Suzhou, China; c Institute of Clinical Medicine Research, Suzhou Hospital, Affiliated Hospital of Medical School, Nanjing University, Suzhou, Jiangsu, China; d Department of Neurology, The Second People's Hospital of Changzhou, The Third Affiliated Hospital of Nanjing Medical University, Changzhou, 213000, China

**Keywords:** Procalcitonin detection, High-affinity antibody, Immunofluorescence lateral flow assay

## Abstract

**Background and aims:**

Procalcitonin (PCT) is a widely recognized inflammation marker utilized in various clinical testing contexts and is subject to ongoing refinements, thereby imposing greater demands on core antibodies. However, the published literature lacks a comprehensive description of them.

**Material and methods:**

In this study, we initially expressed the full-length PCT protein in eukaryotic systems, followed by conventional antibody engineering techniques for *in vitro* cell fusion and the selection of positive hybridoma cell clones specific to the PCT protein.

**Results:**

A total of 83 positive clones were generated, among which 15 high-affinity IgG_1_ subtype monoclonal antibodies were selected for complementarity-determining region (CDR) analysis to predict potential antigen-recognition epitopes. We provided a detailed characterization of the binding properties between these high-affinity antibodies and the PCT protein. Utilizing time-resolved fluorescent microsphere (TRFM), a novel fluorescent microsphere-based immunochromatographic strip (FM-ICS) approach was developed, with MomAb-8 serving as the capture antibody and MomAb-20 functioning as the labeling antibody. Ultimately, following the preliminary evaluation of clinical samples, it was demonstrated that this FM-ICS exhibited a favorable linear range, stability, and clinical relevance.

**Conclusion:**

This study presents a novel approach to enhancing the efficiency of antibody screening across a diverse array of combinations. Furthermore, the method established herein holds significant potential for clinical application in detecting PCT protein using FM-ICS.

## Highlights

• High-Affinity Antibody Generation: Developed 83 monoclonal antibodies against procalcitonin (PCT), selecting 15 high-affinity IgG1 antibodies.
• Novel Detection Method: Created a fluorescent microsphere immunochromatographic strip (FM-ICS) for PCT detection with excellent sensitivity.
• Clinical Sensitivity: FM-ICS demonstrated a detection limit of 1.5 ng/mL and robust performance in clinical sample testing.
• Optimization: Structural analysis and affinity testing improved antibody pair combinations for enhanced detection.
• Application: The method offers potential for infection monitoring and guiding antibiotic use, particularly in sepsis.

## 1 Introduction

Microbial infections and antibiotic resistance rank among the leading causes of morbidity and mortality worldwide. In recent years, procalcitonin (PCT) has gained recognition as a specific biomarker for microbial infections and sepsis, increasingly utilized in the early diagnosis of infections and the monitoring of antibiotic therapy effectiveness [1,2]. PCT is a 116-amino-acid polypeptide precursor of calcitonin, with a molecular weight of 13 kDa [3]. Under physiological conditions, the serum concentration of PCT typically remains below 0.1 μg/L. Serum PCT concentrations ranging from 2 to 10 μg/L are indicative of sepsis, while levels between 10 and 100 μg/L may suggest septic shock [4]. In comparison to other inflammatory markers such as C-reactive protein (CRP), tumor necrosis factor (TNF)-α, and interleukin-6 (IL-6), PCT serves as a more specific and sensitive clinical diagnostic biomarker [[5], [6], [7], [8], [9]]. Nevertheless, with the increasing complexity of pathogenic microorganisms—characterized by the coexistence of multiple bacterial and viral species and the emergence of novel resistant strains—further research is warranted to evaluate the efficacy of methods for detecting serum PCT concentrations.

Several methods exist for detecting PCT levels in blood, including chemiluminescence immunoassay and enzyme-linked immunosorbent assay (ELISA) [[10], [11], [12], [13]]. All of these techniques depend on antibodies exhibiting high sensitivity and specificity. While various novel technology platforms have been developed and successfully implemented—such as phage display technology ^14-17^and flow cytometry-based single-cell sorting combined with sequencing technology [14], however, the development of mouse monoclonal antibody (MomAb) continues to rely on traditional cell fusion techniques. The first specific MomAb targeting PCT was developed in 1988 [15]. In the subsequent years, several additional MomAbs were introduced [16,17]; however, a comprehensive description of their binding characteristics to PCT remains lacking.

In this study, we initially expressed the full-length PCT protein in eukaryotic systems and subsequently utilized it as an immunogen for the selection of positive hybridoma cells. Following the acquisition of a series of monoclonal antibodies, we provided a detailed account of how the antibody combinations employed in the FM-ICS approach were selected, encompassing affinity assessment, sequence analysis of the CDR regions, prediction of potential antigen-binding epitopes, and application of colloidal gold-based immunochromatographic assay (ICA) methods. It is worth noting that the FM-ICS method developed in this study exhibits superior performance in terms of clinical sensitivity (with a detection limit of 1.5 ng/mL) and stability. Moreover, this method offers additional advantages such as reduced assay time and enhanced operational simplicity, thereby providing significant potential for infection monitoring and guiding antibiotic usage.

## 2 Materials and methods

### 2.1 Mice and reagents

Female Balb/c mice were purchased from SiPeiFu Biotechnology (Beijing, China) and housed in standard cages at 24 °C and under 12 h light–dark cycle with ad libitum access to water and food. Animal experiments were approved by The Institutional Animal Care and Use Committee of Experimental Animal Center of Suzhou Institute of Biomedical Engineering and Technology, Chinese Academy of Sciences. A total of 173 serum samples were obtained from patients diagnosed with infections at Suzhou Hospital, Affiliated Hospital of Medical School, Nanjing University, alongside serum samples from healthy controls. Unless otherwise specified, all reagents utilized for cell culture were procured from Gibco, while other chemical reagents were sourced from Aladdin Industrial Corporation (Shanghai, China) or Shanghai Sangon Biotech. HEK293F cells were cultured in FreeStyle™ 293 Expression Medium, and 293T cells and sp2/0 cells were cultured in DMEM with 10 % FBS.

### 2.2 Preparation of recombinant PCT (rPCT) protein

The DNA sequence encoding the N-terminally His-tagged PCT (NP_001365878.1), optimized for expression in HEK293 cells, was synthesized and subsequently cloned into the TK-PCDH-copGFP-T2A-Puro vector. Following this, the vector was co-transfected with packaging vectors into 293T cells to facilitate lentivirus production. Briefly, the envelope vector pMD2.G and the packaging vector psPAX2 were co-transfected with the expression vector into 293T cells using Mirus transfection reagent. The resulting packaged virus was harvested 72 h post-transfection and subsequently utilized for a second round of transfection into HEK293F cells. Following viral transduction, HEK293F cells were cultured in puromycin (4 μg/mL) for 14 days to select for stable rPCT-expressing clones in a 37 °C CO_2_ incubator. The recombinant PCT-His fusion protein was purified via affinity chromatography utilizing nickel-nitrilotriacetic acid (Ni-NTA) spin columns. To identify the protein, sodium dodecyl sulfate–polyacrylamide gel electrophoresis (SDS-PAGE) was conducted using a vertical Mini-PROTEAN Tetra cell apparatus (Bio-Rad, Irvine, CA, USA). The molecular weight of the target protein was determined through Coomassie Blue staining. Additionally, rPCT was analyzed by a commercial ELISA kit (Fintest, Wuhan Fine Biotech Co., Ltd.).

### 2.3 Generation of rPCT-specific hybridomas

Five six-week-old Balb/c mice were immunized with the recombinant antigen, as illustrated in Fig. 2a. The experimental protocol received approval from the Institutional Review Board. Two weeks following the final immunization, blood samples were collected to assess serum titers using ELISA. The mouse exhibiting the highest titer received the final booster immunization via intraperitoneal injection. The mice were euthanized using anesthesia, after which the spleen and lymph nodes were harvested. The purification of B cells was performed by using mouse CD19^+^ B cells isolation kit (StemCell Technologies) according to manufacturer's protocol. Enrichment B cells were subsequently fused with Sp2/0, a murine myeloma cell line, utilizing the CFB16-FB Cell electrofusion device (BEX Co., Ltd., Japan). To selectively culture the hybrid cells, hypoxanthine-aminopterin-thymidine (HAT) medium (Sigma-Aldrich) was employed. A 96-well plate was prepared and filled with 200 μL per well before incubation at 37 °C in a CO_2_ incubator. The cultured hybridomas were screened using an indirect ELISA. To assess the extent of color development, absorbance was measured at 450 nm. Fused cells exhibiting high absorbance were subsequently selected and dispensed into a 96-well plate for serial dilutions. Ultimately, candidate clones were identified through ELISA.

### 2.4 Determination of the binding affinity of MomAbs

For the binding affinity of MomAbs, competitive ELISA was used. 1 μg/mL recombinant PCT protein (in-house expressed) was coated onto the ELISA plates using carbonate buffer (pH-9.5) was kept overnight at 4 °C. Subsequently, coated plates were washed in PBST (1X PBS +0.05 % Tween-20) and blocked in 3 % BSA prepared in PBST for 3 h at 37 °C. MomAb at 1/2 dilution (initial concentration of 0.1 mg mL ^−1^)was pre-incubated with recombinant PCT (50ng/well) for 10min at 37 °C in 100 μl volume. The mix was then added to rPCT coated plates and incubated for an additional 1 h at 37 °C. After washing the plates with PBST, (HRP)-labeled goat anti-mouse IgG was added at 1/4000 dilution for 45 min incubated at 37 °C. The next step of washing was done in PBST and TMB substrate was added for color development. The stop solution was added to stop the reaction followed by absorbance measurement at 450 nm using a microplate reader. Inhibition ability was shown as percentage.

### 2.5 Generation of anti-PCT antibodies

The selected hybridomas consistently produced anti-PCT antibodies in a hybridoma expression medium maintained at 37 °C in a CO_2_ incubator. The supernatant was collected, and the anti-PCT antibodies were purified via affinity chromatography using protein A Sepharose. To assess the purity of the isolated antibodies, SDS-PAGE analysis was performed.

### 2.6 Monoclonal antibody modeling and structural analysis

The cDNA corresponding to the light (VL) and heavy (VH) variable regions of the antibodies was synthesized via PCR from RNA extracted from hybridoma cells. For antibody modeling, the VH and VL sequences were input into the antibody homology modeling software Discovery Studio (Discovery Studio v19.1.0.18287). The VH and VL sequences were individually subjected to BLAST analysis to identify optimal templates in the Protein Data Bank (PDB, https://www.rcsb.org/). Following complementarity-determining region (CDR) loop optimization and energy minimization, the validity of the modeling structure was evaluated using Procheck(https://servicesn.mbi.ucla.edu/PROCHECK/), Profile-3D, and PROSA (https://prosa.services.came.sbg.ac.at/). The binding interactions between PCT and candidate clonal antibodies were analyzed through rigid body docking with the ZDOCK program. An optimized pose exhibiting a high ZDOCK score (>12) was generated utilizing the CHARMm Polar H force field and subsequently refined via the RDOCK program. Ultimately, we selected binding poses based on both RDOCK scores and protein-protein interaction interfaces.

### 2.7 Gold nanoparticle-based monoclonal antibody pair test

Gold conjugates were employed to label antibody binding in a clear and comprehensible manner. Gold nanoparticle-labeled antibodies were applied at a concentration of 20 μg/mL for pairwise testing. Subsequently, the recombinant PCT antigen was diluted in a gold nanoparticle condensation solution and dispensed at a concentration of 10 ng/mL. At this stage, candidate clonal antibodies were applied at 1 mg/mL and sprayed onto the NC membrane. Detection antibodies that recognize epitopes distinct from those recognized by the capture antibody are considered successfully paired antibodies. These antibodies produce a visible color signal on the NC membrane, whereas those failing to bind their corresponding epitopes do not generate any color (Fig. 3a). Thereafter, the optimal matching antibody pairs underwent further evaluation of their analytical performance.

### 2.8 Preparation of fluorescent microsphere (FM) ICA

A fluorescent FM-ICS were developed by integrating TRFM with a lateral flow assay. The use of TRFM for antibody labeling facilitates a clear understanding of the binding process. Briefly, TRFM(0.2 μm,Thermo Fisher Scientific)were suspended in an activation buffer (50 mmol/L MES, pH = 5.0) to prepare a 5 mg/mL microsphere solution. Subsequently, 200 mM EDC and 200 mM NHS were added, and the mixture was allowed to oscillate and activate at room temperature for 1 h after thorough mixing. Following centrifugation at 14,000 rpm for 10 min at 4 °C, the supernatant was discarded, and the resulting pellet was resuspended in triplicate with 200 μL of coupling buffer (0.05 M EMS, pH = 6.0). Subsequently, 1 mL of coupling buffer was added to the tube to resuspend the TRFM, and the resulting solution was divided into ten equal-volume microcentrifuge tubes. In each tube, 10 μg of anti-PCT antibody was introduced and allowed to oscillate and couple at 37 °C for 1 h. Following this incubation, 2 μL of a 20 % BSA solution was incorporated into the suspension, which was then thoroughly mixed and left to oscillate and seal overnight at room temperature. The suspension was carefully removed from the supernatant following centrifugation at 14,000 rpm for 10 min at 4 °C and subsequently re-suspended in a storage buffer consisting of PB buffer with 1 % NaN_3_ and 1 % BSA at pH = 7.4. The microspheres were then washed once using this method and stored in the dark at 4 °C after thorough mixing. The capture antibody was prepared at a concentration of 1 mg/mL and applied to the NC membrane, while chicken IgY was immobilized on the C line as a positive control. Subsequently, labeled IgG antibodies against PCT and labeled goat anti-chicken IgY antibodies were combined and deposited onto a glass fiber membrane to create a marker pad.

## 3 Results

### 3.1 Generation of rPCT in eukaryotic expression system

**Fig. 1:**
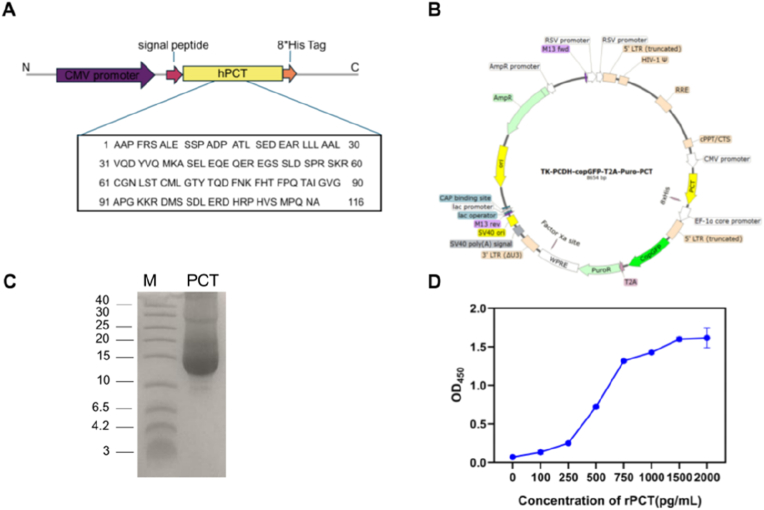
Characterization of rPCT protein. (A) The amino acid sequence of hPCT. (B) The schematics of protein expression vector. (C) An analysis was conducted on the rPCT protein using SDS-PAGE. The 13 kD recombinant protein is visible. (D) The rPCT activity was measured using a commercial ELISA kit.

To achieve a human PCT protein structure that more closely resembles its native conformation, we opted for a eukaryotic expression system. we initially verified the amino acid sequence of this protein using data from NCBI (Fig. 1A), subsequently, we employed gene editing techniques to incorporate this nucleotide sequence into a lentiviral expression vector tagged with His protein, as illustrated in Fig. 1B. Next, an expression vector encoding the human PCT-His gene was transduced into HEK293F cells. Following multiple rounds of *in vitro* drug screening, we successfully established stable cell clones that expressed the target protein. We maximized the collection of cell supernatant and eliminated cellular debris prior to purifying the rPCT-His fusion protein from the culture supernatant using Ni-NTA spin columns. The results showed that a prominent protein band with a molecular weight of ∼13 kDa was detected following SDS-polyacrylamide gel electrophoresis (Fig. 1C). To further validate the reactivity of the expressed protein, we employed a commercial ELISA kit for assessment, and the results indicated that the rPCT protein produced in eukaryotic cells exhibited robust reactivity (Fig. 1D), indicating that the protein may serve as a valuable resource for enhancing animal immunity and facilitating the preparation of hybridoma cells.

**Fig. 2:**
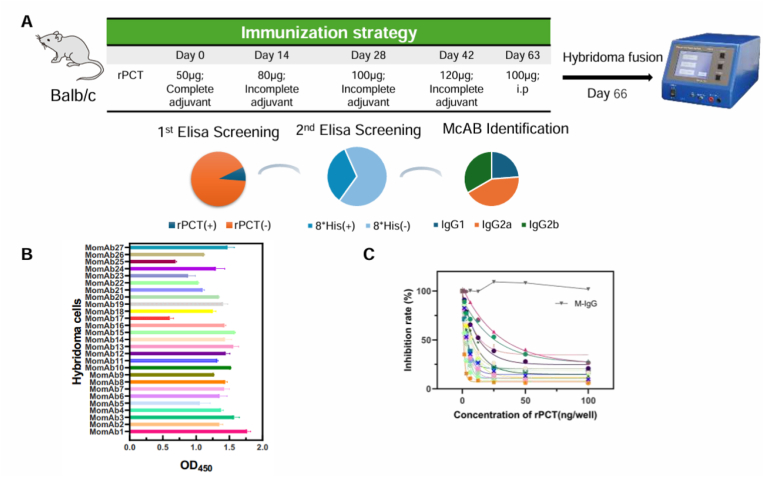
Hybridoma Screening and characterization of MomAbs. (A) Immunization regimens and hybridoma screening statistics. (B) Indirect ELISA for monoclonal antibody screening. (C) The binding affinity of MomAbs to rPCT protein was assessed using a competitive ELISA method. The concentration of MomAbs corresponding to an optical density (OD) of 50 % was utilized to calculate the affinity constant. Mouse endogenous IgG antibody served as the control.

**Fig. 3:**
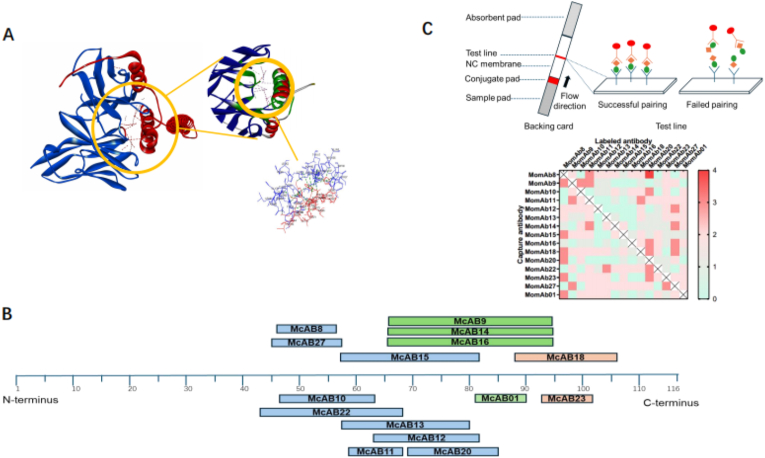
MomAbs analysis and pair test. (A) The docked complex of the predicted MomAb (MomAb-9, blue) and PCT (red) illustrates the interacting amino acid residues along with their respective positions (green). (B) Predicted antigen-binding epitopes mapping(Discovery Studio v19.1.0.18287). (C) Gold nanoparticle-based monoclonal antibody pair testing statistics and the heat map of MomAbs pair test. A darker red color corresponds to greater antibody reactivity towards antigens.

### 3.2 High-affinity anti-rPCT hybridoma screening

Initially, rPCT protein was mixed with Freund's adjuvant and administered to mice through intraperitoneal injection. Following four immunizations, mice exhibiting elevated serum antibody titers were selected for the enrichment of their splenic B lymphocytes. Subsequently, an *in vitro* fusion experiment was performed utilizing the electrochemical fusion. The comprehensive strategy for the selection of high-affinity antibodies involves an initial screening of hybridoma cells capable of recognizing rPCT protein through indirect ELISA assays, while systematically excluding hybridoma cells that exhibit reactivity with the tag protein. This is followed by a minimum of two rounds of single-clonal hybridoma cell culture prior to antibody identification, ultimately retaining those hybridoma cells that are proficient in producing IgG subclasses (Fig. 2A). The results indicated that a total of 783 hybridoma cells were generated following the initial cell fusion, among which 117 cells (15 %) exhibited reactivity with rPCT protein. Notably, 83 of these hybridoma cells (70.9 %) did not demonstrate cross-reactivity with His-tag. Antibody characterization revealed that all selected hybridoma cells belonged to the IgG subclass, comprising 27 antibodies of the IgG_1_ subtype, 20 antibodies of the IgG_2a_ subtype, and 36 antibodies of the IgG_2b_ subtype.

Next, we focus on the IgG_1_ subtype targeting rPCT and systematically assigned sequential numbers to these MomAbs. The majority of antibodies derived from hybridoma cell supernatants exhibited strong reactivity with the rPCT protein, which served as a critical criterion for the selection of high-affinity antibodies (Fig. 2B). We hypothesize that the affinity of antibodies is pivotal in the chromatography reaction system, particularly within the context of the double antibody sandwich method. To develop high-sensitivity detection strips, we employed a competitive ELISA (cELISA) to evaluate antibody affinities. Our findings revealed that 12 antibodies displayed weak to medium affinity, while 15 demonstrated high affinity, including MomAb-8, -9, -18, and −20 (Fig. 2C). These affinity metrics offer valuable insights for selecting appropriate counteracting antibodies in subsequent stages of our research.

### 3.3 MomAbs pair test based on their structural analysis

Subsequently, we investigated the characteristics of these high-affinity antibodies in their recognition of antigens to facilitate the establishment of optimal matching combinations. Initially, we extracted cDNA corresponding to the variable regions of the antibody VL and VH using universal primers, followed by cloning into a T vector for nucleotide sequencing. The percent identities of the variable genes were 58.22 % for the heavy chain and 87.38 % for the light chain. We next investigated whether antibody sequence diversity correlated with epitope diversity for these MomAbs. To further validate the antibody recognition sites, we conducted docking simulations between antibodies and antigens. The homology modeling 3-D structure of MomAb (e.g., MomAb-9) and PCT were shown in Fig. 3B. The results demonstrated the utilization of a diverse array of immunoglobulin V genes throughout the entire panel. More than half of the antibody recognition sites are situated in proximity to the C-terminus of the rPCT protein, whereas only a limited number of recognition signals have been identified near the N-terminus.

We further analyzed the epitopes recognized by MomAb-9, -14, −16, and MomAb-10, as well as the amino acid residues implicated in antigen binding. The three-dimensional homology models revealed significant parallels between the rPCT/MomAb-9 complex and the rPCT/MomAb-14 or -16 complexes, while demonstrating a distinctly different spatial conformation in comparison to the rPCT/MomAb-10 complex (Fig. 3C). The predicted binding sites indicate that these MomAbs may demonstrate varying degrees of steric hindrance, suggesting their potential to form selective combinations exclusively in practical applications. To validate this hypothesis, we subsequently conducted a series of pairwise tests on a chromatographic strip to evaluate each of the 15 candidates MomAbs as both capture antibodies bound to the NC membrane and labeling antibodies utilizing gold nanoparticles. The results revealed that a total of 156 antibody pairs effectively recognized the rPCT protein, as depicted in Fig. 3C, the color intensity within each box corresponds to the mean reactivity of the pairs against individual antigens. It is evident that MomAb-9 is incompatible with either MomAb-14 or -16 for the formation of effective combinations. In contrast, it demonstrated well outcomes when combined with MomAb-8, -10, and −11, which aligns with the analysis of antigenic sites.

This finding indicates that affinity analysis can elucidate the sensitivity of antibodies to antigens, while an examination of binding sites can effectively eliminate ineffective combinations arising from spatial hindrance, thereby enhancing the efficiency of accurately identifying the optimal antibody combination.

### 3.4 Evaluation of methodological performance in the FM-ICS

**Fig. 4:**
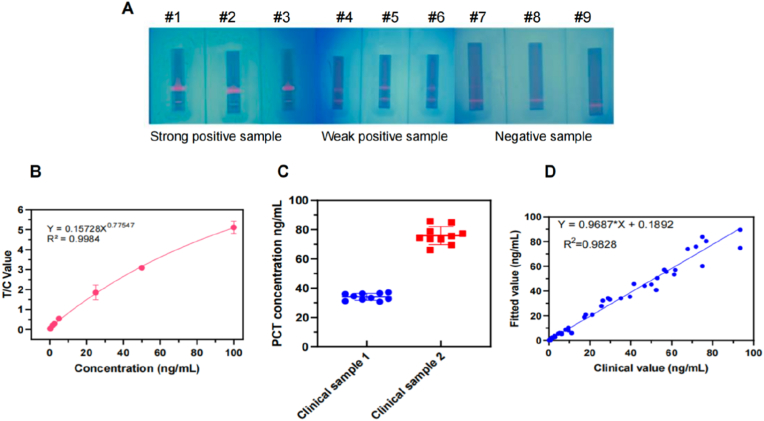
Evaluation of FM-ICS based on MomAb-8 and MomAb-20. (A) Images of the strong/weak positive or negative clinical samples testing by FM-ICS. Statistical chart of linear range(B), stability (C, n = 10), and clinical relevance (D, n = 136) in clinical samples testing using FM-ICS.

Based on our research, when MomAb-8 serves as the capture antibody and MomAb-20 acts as the labeling antibody, the colloidal gold-based ICA exhibits the highest reactivity. Hence, we assessed whether this antibody combination is applicable to FM-ICS. A novel ICA system was developed in which MomAb-20 was labeled onto TRFM in conjunction with MomAb-8. To assess the methodological features of this system, positive PCT samples derived from the serum of clinical patients were examined. Serum from healthy individuals was employed as the negative control. It was found that, in light of the visualizable property of TRFM under ultraviolet light, positive samples could be conspicuously detected and exhibited a favorable gradient compared with the control samples (Fig. 4A).

The linear range, stability, sensitivity, and specificity are critical parameters for evaluating methodological performance. Subsequently, positive PCT samples with known concentrations were serially diluted in a gradient manner to systematically assess the performance of the FM-ICS method developed in this study. The results indicated that the lowest limit of detection was 1.5 ng/mL, demonstrating the method's excellent sensitivity. Conversely, the upper detection limit was established at 100 ng/mL, reflecting a broad dynamic range. Notably, this method exhibited robust stability throughout the entire detection range (Fig. 4B). Weak positive and strong positive samples were selected for measurement, with each sample being assessed 10 times. The repeatability of the weak positive samples was found to be 7.093 %, while that of the strong positive samples was 8.54 %, indicating that this ICA exhibits good stability (Fig. 4C). To further assess the accuracy of the FM-ICS method developed in this study, we tested and analyzed a total of 136 clinical samples stratified into three groups (<0.1 μg/L, 0.1–2.0 μg/L, and >2.0 μg/L), encompassing the full diagnostic threshold range for PCT. The results demonstrated that, compared with the Roche detection platform, our method exhibited excellent correlation (R^2^ = 0.9828 for serum samples), with a regression equation of y = 0.9687x + 0.1892. The results indicated that the serum samples analyzed exhibited similarities to those identified by commercially available imported kits, thereby demonstrating high accuracy and considerable potential for clinical application (Fig. 4D).

## 4 Discussion

In the laboratory, the selection of antibody pairs for layered affinity chromatography is a labor-intensive process. In this study, we employed traditional hybridoma technology to generate a series of specific antibodies targeting the PCT protein. Rather than conducting direct pairing experiments, we prioritized antibodies with potential applications by performing affinity analyses and predicting antigen epitope interactions, subsequently validating them through colloidal gold-based ICA. This approach may be advantageous for identifying suitable pairing antibodies from extensive antibody libraries.

In recent years, TRFM-based ICA have garnered increasing attention due to their high sensitivity, sustained stability, and broad detection linear range [18,19]. Consequently, the development of an FM-ICS method for the quantitative detection of PCT is warranted and may hold significant implications for monitoring secondary infections or assessing the efficacy of antibiotic therapies. However, implementing this approach typically requires the formation of a sandwich complex involving two distinct antibodies that specifically target the antigen. This necessitates that these antibodies do not exhibit overlapping or interfering spatial epitopes when binding to the antigen. Through the application of a suite of bioinformatics analysis tools, we successfully identified the potential sequences of both the heavy and light chains within the antibody variable regions, as well as their probable antigen-binding sites. In this study, the 15 high-affinity antibodies were analyzed following CDR region sequence analysis and structural modeling. The results indicated that the recognition sites of these antibodies predominantly localized to the C-terminal region of the PCT protein, suggesting that a greater number of antigenic sites may be exposed in this area or that these dominant epitopes exhibit enhanced antigenicity. In specific scenarios—such as when the target protein has a small molecular weight, when antibody recognition requires coverage of both N-terminal and C-terminal regions, or when significant spatial steric hindrance is present—it is advisable to employ segmented expression as a strategy for antibody screening.

In Fig. 3C, for instance, the epitope prediction results indicate that MomAb-9 is spatially incompatible with MomAb-14 and MomAb-16, a finding of particular significance. This hypothesis was corroborated by colloidal gold-based ICA, which confirmed that these antibodies cannot form an effective combination. Theoretically, MomAb-9 should be able to combine with other epitope-recognizing antibodies aside from MomAb-14 and MomAb-16; however, experimental results reveal that only combinations of MomAb-9 with MomAb-8, MomAb-10, and MomAb-11 effectively recognize the PCT protein. The underlying mechanism remains elusive.

Affinity assessment is a critical method for selecting potential antibodies; however, our findings indicate that the absolute levels of antibody affinity do not directly determine the efficacy of ICA. This may be due to the fact that affinity is not the sole limiting factor when utilized as either a capture or labeling antibody. Previous studies have reported that dynamic characteristics of antibodies, specifically the equilibrium dissociation rate during antigen recognition, are also significant influencing factors in ICA reactions [13]. In this study, we employed the optimal antibody identified from colloidal gold-based ICA for validation in FM-ICS; however, it remains unclear whether there exists a complete correspondence between these two systems despite both being immunoassay methods. Notably, for labeling antibodies, the principles governing tracer binding differ: colloidal gold is generally considered to bind non-covalently to antibodies, whereas TRFM is believed to engage through covalent bonds [20,21]. Addressing this discrepancy will constitute an important scientific question for future investigation.

## CRediT authorship contribution statement

**Xiaoxia Cheng:** Writing – review & editing, Writing – original draft, Visualization, Validation, Supervision, Software, Resources, Project administration, Methodology, Investigation, Funding acquisition, Formal analysis, Data curation, Conceptualization. **Lichen Zha:** Writing – review & editing, Writing – original draft, Visualization, Validation, Methodology, Investigation, Funding acquisition, Formal analysis, Data curation, Conceptualization. **Jiao Yang:** Visualization, Validation, Supervision. **Yinyin Qin:** Supervision, Software. **Ruhong Yan:** Validation, Supervision, Resources. **Yuzhu Ma:** Visualization, Supervision. **Changsong Zhang:** Visualization, Validation, Supervision. **Hongran Fu:** Writing – review & editing, Methodology, Investigation, Funding acquisition, Formal analysis, Data curation, Conceptualization.

## Ethics approval and consent to participate

All mouse experiments were performed according to the Guidelines for the Care and Use of Laboratory Animals (2017 version, issued by the Ministry of Health, People's Republic of China). The Institutional Animal Care and Use Committee of Experimental Animal Center of Suzhou Institute of Biomedical Engineering and Technology, Chinese Academy of Sciences approved these studies.

This study was approved (or granted exemption) by the medical ethics committee of Suzhou science and technology town hospital (approval no.IRB2024065). We certify that the study was performed in accordance with the 1964 declaration of HELSINKI and later amendments.

## Consent for publication

Written informed consent for publication was obtained from all participants.

## Availability of data and material

The authors confirm that the data supporting the findings of this study are available within the article and its supplementary materials.

## Funding

This study was supported by Suzhou Science and Technology Bureau (KJXW2021088, SKYD2022090), General Nanjing Medical University of the Technology Development Fund (grant no. NMUB20210254),Suzhou Municipal Commission of Health and Family Planning (grant no. LCZX202031) and the 10.13039/501100010881Suzhou Science and Technology Program (grant no. SLT202005).

## Declaration of competing interest

The authors declare that they have no known competing financial interests or personal relationships that could have appeared to influence the work reported in this paper.

## Data Availability

Data will be made available on request.
