# Supplementary material for: The quantitative detection method employing a combination of high-affinity antibodies targeting PCT
Source: Biochem Biophys Rep. 2025 Jun 30;43:102091. doi: 10.1016/j.bbrep.2025.102091 (PMC12270020; doi:10.1016/j.bbrep.2025.102091)
Supplement: Multimedia component 1 [file mmc1.docx]

Clarification on the Use of Female Balb/c Mice in the Study

Dear Editor/Reviewers,

Thank you for reviewing our manuscript and for your valuable feedback. Regarding your concerns about the selection of animal sex in our study, we provide the following clarifications:

This study employed female Balb/c mice based on:

Model Maturity: Balb/c mice are a standardized strain widely used in immunology and oncology. Female individuals exhibit stable physiological traits (e.g., baseline immune responses), minimizing inter-individual variability.

Alignment with Research Objectives: In addressing the question of mouse gender and its potential influence, we would like to share that in this study, we exclusively used female mice. One reason for this choice is practical: female mice tend to be much less aggressive than males, which minimizes stress or injuries from fighting and helps maintain a stable immune process. Additionally, research has shown that female animals, thanks to the presence of estrogen, often demonstrate stronger humoral immune responses compared to their male counterparts. This characteristic may enhance the likelihood of successfully obtaining high-quality hybridoma cells. We have incorporated these considerations into an updated description within the original text. Existing literature confirms no significant sex-related impact on core metrics in this context^[1,2]^.

We appreciate your review and are open to incorporating any additional information as per your recommendations.

References

[1]	Gao Y, Sang F F, Meng D L, et al. Preparation of a novel monoclonal antibody against caprine interleukin-17A and its applications in immunofluorescence and immunohistochemistry assays[J]. BMC Biotechnology, 2019, 19(1): 47.

[2]	Fernandes Fidelis C, Silva De Araújo L, Prates-Patarroyo P A, et al. Immunisation with neospora caninum subunits rsNcSAG4 and rsNcGRA1 (NcSAG4 and NcGRA1 epitopes construct) in BALB/c mice: the profile of the immune response and controlling the vertical transmission[J]. Parasitology Research, 2024, 123(1): 58.

Sincerely.

Hongran Fu

Department of Neurology, The Second People's Hospital of Changzhou, the Third Affiliated Hospital of Nanjing Medical University, Changzhou 213000, China.

Email: xiaofu6666@163.com
